# The use of low-cost ruggedized Android tablets to augment in-service training of community health workers in Mukono, Uganda: perspectives and lessons learned from the field

**DOI:** 10.4314/ahs.v21i3.60

**Published:** 2021-09

**Authors:** Christina E Stiles, Edward O'Neil, Kenneth Kabali, James O'Donovan

**Affiliations:** 1 Omni Med, Waban MA, USA and Kisoga Town, Mukono District, Uganda; 2 The University of Chicago, Pritzker School of Medicine, Chicago, IL, USA; 3 Steward St Elizabeth's Medical Center, Department of Emergency Medicine, Boston, MA, USA; 4 University of Oxford, Department of Education, Oxford, UK

**Keywords:** Low-cost ruggedized Android tablets, in-service training, community health workers, Mukono

## Abstract

**Background:**

Despite potential for community health workers (CHWs) to effectively reduce morbidity and mortality in sub-Saharan Africa, they still face multiple barriers including access to on-going and refresher training. Digital technology offers a potential solution to improve the provision of ongoing training for CHWs.

**Objectives:**

This report shares participant insights and experiences following the implementation of a mobile health (mHealth) assisted Integrated Community Case Management (iCCM) refresher training programme for CHWs in Mukono, Uganda. We seek to document benefits and challenges of such an approach.

**Methods:**

CHWs were trained to recognize, treat and prevent childhood pneumonia via locally made videos preloaded onto low cost, ruggedized Android tablets. Subsequent interviews were compiled with key stakeholders including CHWs, CHW leaders and programme supervisors to better understand the strengths, barriers and lessons learned following the intervention.

**Results:**

Success factors included the establishment of CHW leadership structures, the ability to use the tablets to learn on an “any pace, any place” basis and using the tablets to conduct community teaching and outreach. Barriers included appropriate consideration of the implementation timeline and avoiding a “one size fits all” approach to digital literacy training.

**Conclusions:**

The strength of the program stemmed from a grassroots approach that prioritized stakeholder input at all stages. Leadership at a local level, a history of local engagement and trust built up over a period time were also integral. As organizations aim to scale up digitally enhanced training initiatives, it is paramount that attention is paid to these human factors which are key for program success.

## Introduction

For complex multifactorial reasons, low and middle-income countries (LMICs) are struggling to improve population health outcomes^1^,^2^. Training community health workers (CHWs) and deploying them within their communities has been suggested as one way to help address the shortage of trained health care professionals and deal with major health concerns such as diarrhoea, malaria and pneumonia (DMP) 3–7. CHWs are defined as lay workers who live in the area they serve, are based primarily in the community, perform tasks related to healthcare delivery and have received some organised training, but may not hold a formal qualifications^8^.

CHWs are uniquely placed to address some of the major health burdens facing their communities given that they are serving the locality where they live and therefore understand the socio-cultural sensitivities that influence health behaviours^9^,^10^. Furthermore, CHWs are often well-respected members of their communities and have the unique potential to serve as a broker between the formal and informal community health sectors^11^.

CHWs programs have proven to be effective at reducing morbidity and mortality from diseases such as DMP, yet major challenges around financial remuneration, retention and provision of ongoing training remain^12^–^14^. In particular, a lack of coordinated on-going refresher training has been suggested as one contributing factor to the failure of CHW programs over time^15^. Recent advances in technology offer one potential solution to help address this challenge^16^–^19^.

In 2002, Uganda began implementing a CHW program in which lay members of the community were elected to serve as Village Health Teams (VHTs)^20^. VHTs are defined by the Ugandan Ministry of Health (MoH) as community volunteers elected by their communities and given basic health training^21^. The aims of the program are to “mobilize and sensitize communities to actively participate in utilizing available health services” via education, preventive health measures, referring sick patients to local health centres, and “community disease surveillance through active data collection and reporting” 14,21. The training, recruitment and ongoing support of VHTs is conducted primarily by non-governmental organisations (NGOs), which are endorsed by the government at a local, district and/or national level^13^,^22^. One such organization is Omni Med (OM), a Ugandan NGO which has worked in partnership with the MoH since 2008 to train, maintain and engage VHTs across the Mukono district^23^. VHTs who were involved in the study will be referred to hereafter as CHWs. CHW leaders indicates locally elected leadership while CHW supervisors refers to the OM staff who oversaw program implementation.

In 2012, Integrated Community Case Management (iCCM) was introduced in Uganda to train CHWs in the prevention, early detection, and treatment of diseases such as DMP which represent the leading causes of morbidity and mortality in children under five in Uganda^21^,^24^. iCCM training is traditionally delivered as a five-day course in one location taught didactically by a CHW supervisor. One-day refresher trainings are then delivered every six months^21^. Despite this, a study in Uganda found that a lack of refresher trainings was a major challenge faced by CHWs, and trainings were often not delivered as scheduled^14^.

In early 2017, OM piloted a mobile health (mHealth) training program to provide alternatives to conduct refresher trainings. Instructional videos of an OM staff member presenting adapted iCCM material were uploaded onto low cost Android tablets. These tablets were subsequently used to train CHWs in accordance with iCCM guidelines to recognize, treat and prevent pneumonia^25^. The educational findings of this pilot randomized control trial (RCT) have been published elsewhere^26^.

The aims of this current study are to present the findings from qualitative interviews with CHWs, CHW leaders and CHW supervisors regarding the benefits and challenges of an mHealth assisted approach to refresher training.

## Methods

Located in central Uganda, the Mukono district is composed of 15 sub-counties, which are divided into 80 parishes and 592 villages^27^. In 2011, pneumonia was reported as the leading cause of death for children under five within the district, with a case fatality rate of five percent^28^. CHWs in the region received initial onboarding by MoH personnel and are overseen inconsistently by various governing bodies including several NGOs.

In brief, CHWs were recruited from the Mpatta, Nakisunga and Mpunge sub-counties of the Mukono District. CHWs were eligible to participate in the study if they had received initial iCCM training but had not participated in any pneumonia specific refresher trainings within two years. Across the three sub-counties, 129 eligible CHWs consented to participate and subsequently completed the study. CHWs were randomised by sub-county, leaving 66 and 63 CHWs in the control and intervention respectively. There were no significant differences between groups in any recorded demographics (age, years as a CHW, gender, average number of children in household)^26^.

Mpatta, Nakisunga and Mpunge sub-counties did not have established leadership structures before the research trial. The MoH does not have any guidelines regarding elected CHWs leadership within the communities, so leadership structures and elections followed local customs with the assistance of the respective village LC1 chairperson. In the weeks preceding the training, CHW leaders were elected in each of the participating parishes (n=7). CHW leaders added a layer of oversight and support for the CHWs and served as the primary liaison between the CHWs and the CHW supervisors.

**Figure 1:**
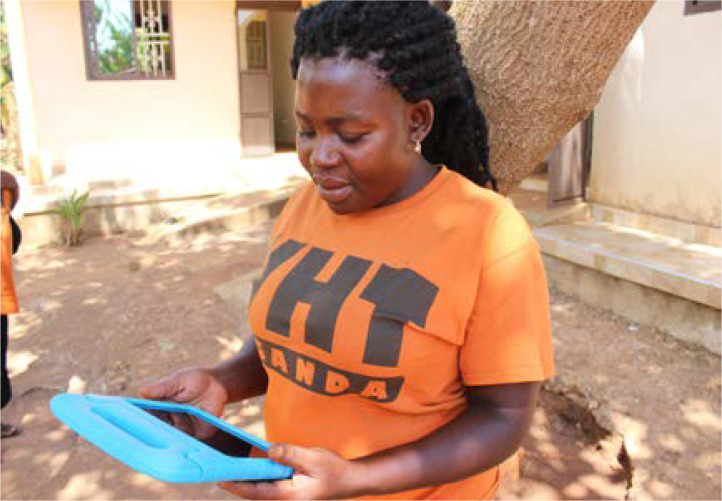
A CHW using a tablet to view the pneumonia videos during the half-day digital literacy workshop with Omni Med staff. Photographer: James O'Donovan.

The CHWs in the control group received a one-day didactic training by OM staff on pneumonia. The CHWs in the intervention group received a half-day digital literacy workshop with an OM staff member on how to use the electronics tablets to watch and share pre-loaded instructional videos (see Figure 1) which they then used for the subsequent six days until they were collected. During these six days, CHWs used the tablets at their discretion, and were linked with the local leaders for assistance and technical support^26^. For a full description of the training, randomization process and intervention protocol, please refer to the corresponding RCT^26^.

**Figure 2:**
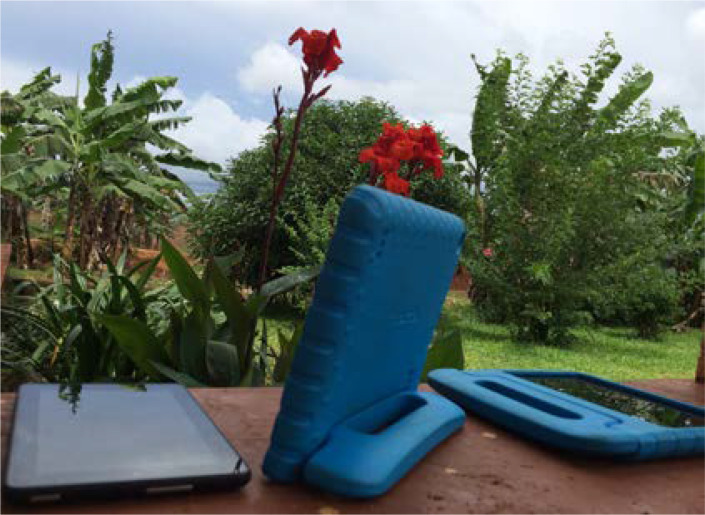
Sample android tablets displayed with and without ruggedized cases. Photographer: Christina Stiles.

Context appropriate technology was used. Amazon Kindle Fire 7 tablets were chosen since they were relatively low cost ($50 USD) and had a two-year repair warranty. They were housed in ruggedized rubber protective cases to shield them from damage (see Figure 2). The tablets had a microSD card slot, allowing videos to be pre-loaded onto the tablets and accessed independent of Internet availability.

**Figure 3:**
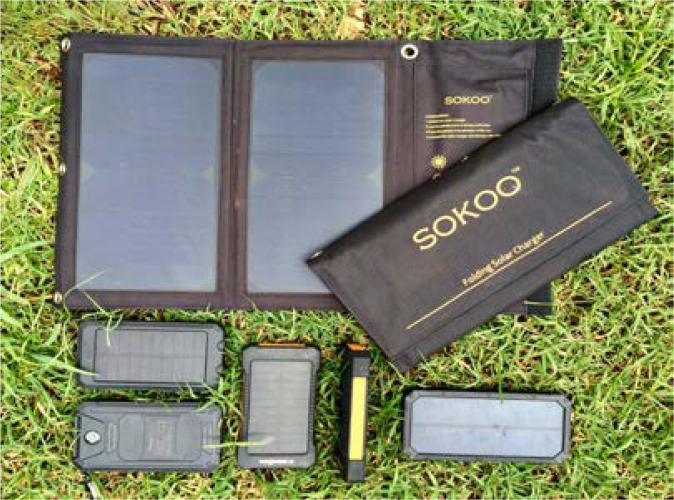
The four different types of solar chargers used by CHWs. Photographer: Christina Stiles.

Four different types of solar chargers were tested during this pilot project (see Figure 3). These were chosen due their low cost, positive reviews, and additional features e.g. flashlights. Specific details regarding the solar chargers can be found in the Appendix.

Purposive sampling was used in order to obtain a range of views and perspectives across different geographical locations and CHW cadres. CHWs, CHW leaders and CHW supervisors were invited to take part via an oral invitation, provided with an information sheet and given two weeks to respond. Participation was voluntary, and all participants signed an informed consent form.

After the tablets were collected, participants were asked to partake in individual semi-structured interviews regarding their experiences being involved in the study. Semi-structured interviews were chosen to allow participants the chance to speak openly about their experiences without being influenced by other participants. Interviews were conducted by two of the study authors in both English and Luganda depending on the preference of the participant. Interviews conducted in Luganda were facilitated by a translator. The interviews with CHWs and CHW leaders took place in their respective villages while interviews with the CHW supervisors took place at the OM office.

All audio files were recorded on a mobile phone or via a handheld digital recording device. In line with the approved ethics protocol, hard copies of the transcribed files were stored in the study master folder which is kept in a locked cabinet within a secure room at the OM office. Data from the audio files was transcribed manually by the authors who conducted the interviews. The same authors independently reviewed and coded the transcripts through content analysis for examples of themes, taking an inductive approach towards analysis. The authors then compared their transcripts and compiled a list of example codes.

## Results

A total of 3 CHW supervisors, 7 CHW leaders and 7 CHWs took part in the semi-structured interviews. These interviews yielded themes addressing the benefits and challenges of utilising tablet technology to assist the delivery of ongoing CHW training.

First, electing local CHW leaders who were able to liaise between CHWs and study staff helped facilitate the success of the study. One of the programme supervisors highlighted this by stating:

“*That was important... getting information from the leaders [about] where a [CHW] is. If a [CHW] is dead, if a [CHW] is no longer interested in the program... at least we have that leader*.” (Female CHW supervisor)

Beyond augmenting the ongoing training of CHWs, the tablets were also used by CHWs to share information with local community members. This is illustrated by the following quote:

“*They [CHWs] can go to their neighbours and... that neighbour can get the information directly as it was told in the video. So, the villagers take real information which is not changed by passing through someone else's mouth*.” (Male CHW supervisor 1)

This trickle-down effect, whereby information is shared at a community level, may be particularly beneficial for education and behaviour change. As smart phone ownership becomes more prevalent in LMICs, CHWs may have the ability to directly share information and resources such as educational videos with community members.

Additionally, by delivering training using locally made, contextually appropriate videos on low cost tablets, CHWs were able to engage in learning on a “any pace, any place” basis. CHWs feedback showed that they find this method of training particularly valuable due to its flexible nature, illustrated below:

“*Some people would say “Oh I lost a relative, that is why I didn't attend” but if we have the information already on the tablet, [it can be used] anytime someone may have free time*.” (Female CHW supervisor)

This program was particularly beneficial for CHWs in Uganda, who serve on a voluntary basis. Outside commitments such as household duties and economic activities can be prioritized while engaging in training at convenient times in a non-formal setting. This flexibility also allows CHWs to revisit content that they do not understand.

“*When you keep all the information in the tablet, if we went anywhere in the community, you can just open it and start training*.” (Male CHW supervisor 2)

Particularly since they serve as volunteers, CHWs and CHW supervisors all emphasise that being aware of competing interests and the livelihoods of CHWs is important. In the context of this study, the timeline of implementation played a role in our outcomes:

“*The [CHWs] took the tablets, but failed to use them because of the hard work cropping, weeding, and when it comes to Friday they fear to [return] because they don't know [the content that] is on the tablet. Others failed to participate because they were busy growing their crops, so it is better to avoid implementing our programs in the planting season*.” (Male CHW supervisor 1)

Basic needs, including food provision, will often take a priority over training events particularly when the service model is based around a voluntary cadre of CHWs. Recognition of the individual needs of CHWs is particularly important to ensure that this form of digitally assisted training does not follow a “one size fits all” model. Many of the older CHWs struggled to read small text on paper and the screens of the tablets.

“*I think... some were complaining because they don't see well, they need reading glasses*” (Male CHW supervisor 2)

Potential solutions include ensuring the text and audio of the tablet are optimised and providing additional in-person support to those CHWs who struggle to use digital technologies.

A CHW centred approach includes ensuring digital literacy is established prior to program commencement. Despite OM staff holding a half-day digital literacy and orientation course, some CHWs felt this was not long enough:

“*The time was [short] for them to learn... to learn the tablet itself and also learn what is on the tablet*.” (Female CHW supervisor)

One of the male supervisors felt the course should have been at least two days in length, since some CHWs called him regarding technical problems, as illustrated below:

“*Some were calling... when they failed to open up the videos or failed to charge... those kinds of things they need like two days to get used to*.” (Male CHW supervisor 1)

CHW supervisors also commented that the digital literacy course needed to be more thorough, so that CHWs felt truly comfortable in using the tablets to their full potential. One supervisor commented that a CHW said: “*You taught us about the videos only, but if something happens to my tablet what [do I] do?*” (Female CHW supervisor)

Ongoing technical support also needs to be considered, and concerns were raised by CHW supervisors regarding this:

“*If they get any mechanical problem do, [CHWs] have a way of fixing them?*” (Female CHW supervisor)

Furthermore, given the narrow focus of this study on assessing the use of instructional videos to enhance CHWs on-going training, we did not make optimal use of the functionalities afforded by tablet-based training, such as the ability of digital technologies to support ongoing supervision and form interactive networks through digital messaging. One of the CHW supervisors stated:

“*We want this [CHW] to be able to use the tablet to either send information to where a patient should go, or ask questions [that are] going... to be answered*.” (Female CHW supervisor)

The same CHW supervisor also highlighted the potential benefits of the tablets for recapping training and highlighting areas they find difficult to understand:

“*Where he has not understood...he can just go back to the videos, to the reading material that are there, read and understand, and if possible even ask questions using the tablets*.” (Female CHW supervisor)

## Discussion

The CHW responses highlight the importance of considering human factors and ensuring contextual relevance in the implementation of a digitally assisted on-going training programme for CHWs in rural Uganda. The RCT comparing the knowledge acquisition of the two groups found that tablet-based training was comparable to traditional training with no statistical difference between the groups, highlighting that digitally assisted training is an exciting tool to implement in LMICs^26^. Given this exciting future, we sought the views of key stakeholders including CHWs, CHW supervisors, and CHW supervisors to understand what contributed to the overall success of this project along with key challenges.

First, strong local community networks and leadership were integral to program success. The establishment of leadership at the local level, combined with OM's sustained presence and grass roots approach, built inherent trust between CHWs and study staff that contributed to the smooth implementation. This intimacy facilitated a better understanding of local context, particularly when considering demographic variation and competing local interests. Indeed, a 2014 comparison between CHW programs in Kenya, one implemented by the MoH and the other by an NGO, found that while both programs faced many challenges, the NGO model was able to introduce key innovations as a result of the flexibility and foresight afforded by working closely on the ground^30^.

Additionally, having local champions to bridge the gap between the implementation team and CHWs enabled effective mobilization and communication with the CHWs. Within the literature, supervision and social support has historically been cited as a principle barrier to CHW program success^13^,^14^,^30^–^32^. Braun et al. noted that the literature contains several examples of tools to improve supervision of CHWs, but few instances of interventions to improve CHW leadership^33^. The success of local leadership as demonstrated by our intervention thus presents a gap in the literature to be further explored. Some even argue that when used appropriately, technology can play a role in both supervision and leadership, as was suggested by our CHWs on the ground^33^. This dual functionality would be an interesting next step for our intervention and those investing in new technologies.

**Figure 4:**
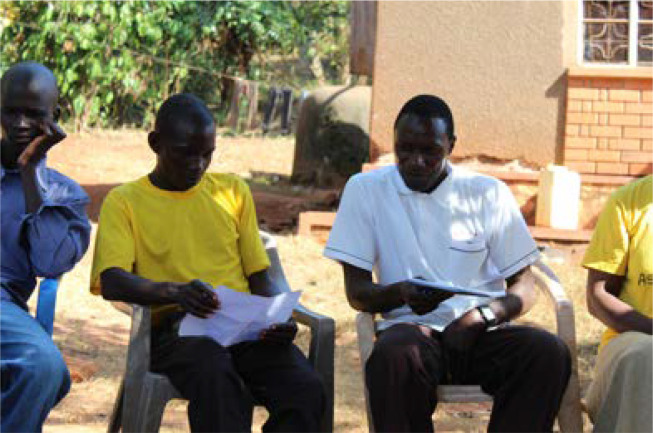
CHWs gather to learn about the implementation of the trial and participate in a group discussion facilitated by Omni Med staff. Photographer: James O'Donovan.

Furthermore, key stakeholder involvement at every stage and from every level (from CHWs on the ground to government officials) is crucial. In the OM program, development of training material was conducted in partnership with local stakeholders. This meant featuring local people in the training videos and ensuring the content was filmed in the local language (Luganda) which helped increase the overall acceptability of the program to the local population (see Figure 4). This stakeholder centred approach shifted the focus towards the needs of the community, which include the flexibility to learn on their own terms and utilize the teaching tools with their neighbours. This is consistent with other published reports highlighting the importance of local engagement and inclusion in the development process for CHW buy in^34^. We also obtained support from the MoH to ensure the adapted iCCM training materials were in line with their policies and informed the District Health Educator of our work. With current ongoing changes to the CHW program in Uganda, involving government level stakeholders will be important to ensure CHWs remain engaged and involved^21^,^22^.

While this study examines the experiences and perceptions of CHWs, it does not explore the same within the surrounding community and local leadership. Further focus group discussins or surveys of the neighbours and community members are therefore needed to understand the effect of the programme at household level. Moreover, it is important to note that the interviews were carried out by OM staff members. This might have biased the responses of some of the participants, however precautions were taken to minimise responder bias.

## Conclusions

With large-scale investments being made in digitally assisted training for CHWs in LMICs, there is a need for organizations responsible for implementing such programs to consider some of the approaches and challenges outlined in this practical report^19^. It is important to ensure equal emphasis is placed on both the human and technical factors of such programs. Local buy in is of paramount importance to ensure success of such projects and should therefore be at the forefront of digital development initiatives. By incorporating local NGOs, researchers have additional resources and knowledge of local customs at their disposal to facilitate stakeholder involvement, local leadership and an understanding of societal context within large scale roll outs.
